# Optimal Design of Sparse Matrix Phased Array Using Simulated Annealing for Volumetric Ultrasonic Imaging with Total Focusing Method

**DOI:** 10.3390/s24061856

**Published:** 2024-03-14

**Authors:** Dmitry Olegovich Dolmatov, Vadim Yurevich Zhvyrblya

**Affiliations:** School of Non-Destructive Testing, National Research Tomsk Polytechnic University, 30 Lenin Avenue, 634050 Tomsk, Russia

**Keywords:** ultrasonic nondestructive testing, ultrasonic imaging, total focusing method, matrix phased arrays, sparse phased arrays, optimization task, sparse array optimization, stochastic optimization methods, simulated annealing

## Abstract

The total focusing method (TFM) is often considered to be the ‘gold standard’ for ultrasonic imaging in the field of nondestructive testing. The use of matrix phased arrays as probes allows for high-resolution volumetric TFM imaging. Conventional TFM imaging involves the use of full matrix capture (FMC) for ultrasonic signals acquisition, but in the case of a matrix phased array, this approach is associated with a huge volume of data to be acquired and processed. This severely limits the frame rate of volumetric imaging with 2D probes and necessitates the use of high-end equipment. Thus, the aim of this research was to develop a novel design method for determining the optimal sparse 2D probe configuration for specific conditions of ultrasonic imaging. The developed approach is based on simulated annealing and involves implementing the solution of the sparse matrix phased array layout optimization problem. In order to implement simulated annealing for the aforementioned task, its parameters were set, the acceptance function was introduced, and the approaches were proposed to compute beam directivity diagrams of sparse matrix phased arrays in TFM imaging. Experimental studies have shown that the proposed approach provides high-quality volumetric imaging with a decrease in data volume of up to 84% compared to that obtained using the FMC data acquisition method.

## 1. Introduction

Phased arrays are increasingly being used in ultrasonic testing because of their flexibility in solving a variety of materials inspection tasks. This type of transducer consists of a number of individual piezoelectronic elements; therefore, different approaches to signal acquisition can be used. One of the approaches is FMC [1], which involves the sequential transmission of ultrasonic waves by each element of the phased array and the reception of the reflected signals by all elements of the probe. The resulting set of signals can then be post-processed to restore the image of the internal structure of the test object. A combination of FMC and post-processing using algorithms based on the synthetic aperture focusing technique was first proposed by Holmes et al. [2] and was referred to as TFM. The combined use of TFM and FMC is often referred to as TFM/FMC, and this approach is widely regarded as the benchmark for ultrasonic imaging in nondestructive testing [3].

Real-time imaging with TFM/FMC can be challenging when large arrays are used and when inspections are to be carried out at high scanning speeds (e.g., railway inspection [4]). This is due to the time required to acquire the signals using the FMC and the time required to restore the image by post-processing the acquired signals. Increasing the speed of TFM imaging is therefore a hot research topic in non-destructive testing. For this purpose, computationally efficient post-processing algorithms can be applied [5,6], and computations within post-processing algorithms could be transferred to field-programmable gate arrays [7,8] or graphics processing units [9,10]. Another approach to increase the speed of TFM imaging is to use sparse arrays [11] when a limited number of elements are used to transmit ultrasonic waves and receive the reflected signals. The speed of imaging performed using sparse arrays is higher compared to the FMC method, since a limited volume of signals to be post-processed reduces the ultrasonic data acquisition time and image restoration time. In addition, a limited number of active elements can reduce the requirements for the electronic units used, thus reducing their cost. Finally, the configuration of the sparse phased array can be varied with respect to the ultrasonic imaging conditions, proving the versatility of this approach.

The key factor that determines the efficiency of ultrasonic imaging using sparse arrays is the determination of their layout (configuration) to obtain a high-quality result. Therefore, several approaches have been proposed to determine the sparse configuration in the case of two-dimensional imaging using TFM and linear phased arrays. In reference [12], Bannouf et al. implemented an iterative procedure to determine the linear sparse array layout. At each iteration, a new element was introduced into the configuration to maximally reduce the side lobe level (SLL) and the main lobe width (MLW) of the point spread function of the sparse array. The iteration process was completed when the point spread function of the sparse phased array reached the specified parameters. Another approach to determine the optimal sparse array configuration is the application of stochastic optimization methods. Hu et al. [13,14] proposed a genetic algorithm for this purpose. The authors introduced the beam directivity function of the sparse array and optimized the parameters of the beam directivity diagram using the genetic algorithm. The genetic algorithm was also used to determine the layout of sparse arrays in reference [15] by Bazulin et al. In this case, the optimization is performed based on the difference between the image obtained using the considered configuration of the sparse phased array and the restored results when FMC is applied. Zhang et al. [16] considered particle swarm optimization to determine the optimal sparse array configuration. Optimization of the beam directivity function parameters was also applied in this research.

Volumetric ultrasonic imaging with matrix phased arrays is another example of an area where real-time acquisition of the results is challenging [6]. Since the pitch of uniform phased arrays should not exceed half a wavelength [11], 2D probes must contain a large number of elements in order to have a sufficient aperture size. This also increases the demands placed on the electronics units used. Similar to the 2D ultrasonic TFM imaging with linear phased arrays, the application of sparse probes can be considered for the volumetric case. Although sparse matrix phased array configuration determination is a hot topic in medical imaging [17,18,19,20,21], it has not been considered in the field of nondestructive testing and TFM imaging. Determination of the sparse array configuration can be regarded as the optimization problem, which can be solved using stochastic optimization methods. On the basis of the research conducted in the medical [19,22,23] and nondestructive testing [13,14,15] fields, two stochastic optimization algorithms have found widespread application: simulated annealing and the genetic algorithm. The performance of these stochastic optimization methods largely depends on their implementation and the problem to be solved, yet simulated annealing is the more highly recommended of these two methods [24].

Thus, the aim of this research was to develop a method for determining the optimal sparse matrix phased array configuration in volumetric TFM imaging. The aforementioned task within the method to be developed was treated as an optimization problem, which was solved using simulated annealing. The method developed based on this approach provides high-quality ultrasonic imaging under various inspection conditions. An effective implementation of simulated annealing for the solution of the sparse matrix phased layout optimization problem requires the introduction of the acceptance function. Within the aforementioned problem, this function should apply the parameters of the acoustic field produced by sparse 2D probes in TFM imaging. This requires the implementation of these approaches to calculate the above parameters. Furthermore, the parameters of simulated annealing must be determined to obtain a fast and global solution of the optimization problem. All the considered issues have not been studied in sufficient detail in the scientific literature in relation to volumetric TFM imaging with matrix phased arrays. This necessitated the research reported in this paper.

## 2. Theory

### 2.1. Beam Directivity Function of Matrix Phased Arrays

The optimum sparse array configuration should be determined with regard to ultrasonic imaging conditions. Consider the data acquisition mode of the matrix phased array layout, which involves sequential emission of ultrasonic waves by each active element of the configuration and recording of reflected acoustic signals by all active elements. This can be either a sparse 2D probe or a full matrix phased array where all the elements of the transducer are active.

**Figure 1:**
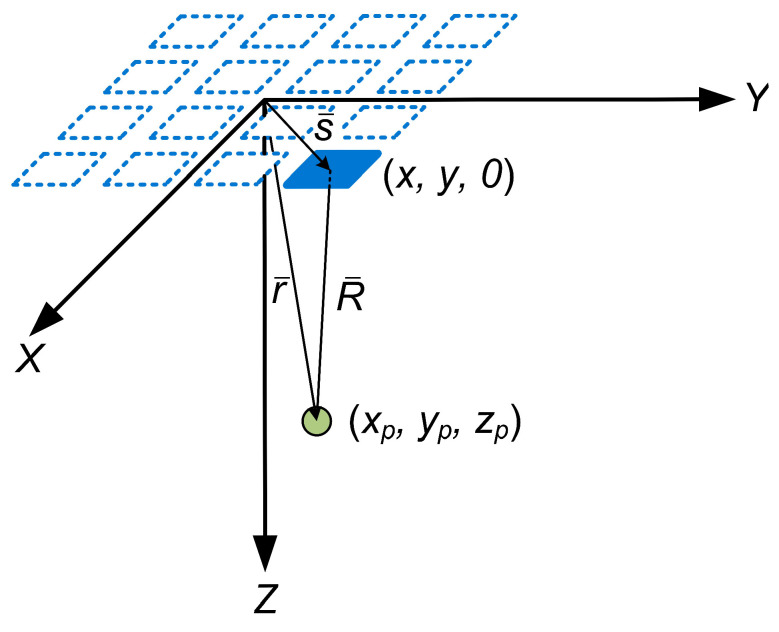
Vectors $\bar{R}$, $\bar{s}$, and $\bar{r}$ for the considered element of the matrix phased array.

Consider an element of the matrix phased array with a center located at a point with coordinates (*x*, *y*, *z* = 0), and consider a point in space with coordinates (*x_p_*, *y_p_*, *z_p_*). Let $\bar{R}$ be the vector from the center of the element to the point in the medium with coordinates (*x_p_*, *y_p_*, *z_p_*), let $\bar{r}$ be the vector from the point in space to the origin of the coordinate axis, and let $\bar{s}$ be the vector from the origin of the coordinate system to the center of the element of the probe (Figure 1).

The law of cosine can be written as follows:

(1) $${\left\|\bar{R}\right\|}^{2}={\left\|\bar{r}\right\|}^{2}+{\left\|\bar{s}\right\|}^{2}-2\cdot \left\|\bar{r}\right\|\cdot \left\|\bar{s}\right\|\cdot \cos(\alpha )$$

where α is the angle between vectors $\bar{r}$ and $\bar{s}$.

In the far field distance, $\left\|\bar{R}\right\|$ is sufficiently large. Thus, $\left\|\bar{R}\right\|$ can be approximated according to reference [25] to the first order as follows:

(2) $$\left\|\bar{R}\right\|\approx \left\|\bar{r}\right\|-\left\|\bar{s}\right\|\cdot \cos(\alpha ),$$

The cosine of angle α can also be expressed as follows:

(3) $$\cos(\alpha )=\frac{x_{p}\cdot x+y_{p}\cdot y+z_{p}\cdot 0}{\left\|\bar{r}\right\|\cdot \left\|\bar{s}\right\|},$$

**Figure 2:**
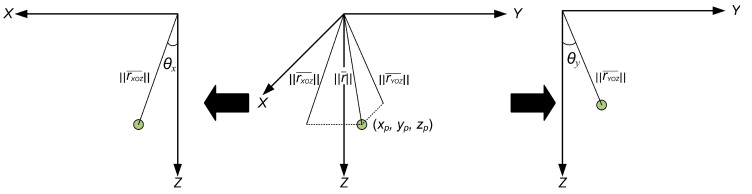
Angles $\theta_{x}$ and $\theta_{y}$ in the considered coordinate system.

Consider the non-conventional spherical coordinate system reported in reference [26]. In this coordinate system, each point is described by two angular coordinates and by the distance between the origin of the coordinate system and the considered point. In this case, angle $\theta_{x}$ describes the angle between the projection of the distance on the XOZ plane and the OZ axis, while angle $\theta_{y}$ describes the angle between the projection of the distance on the YOZ plane and the OZ axis (Figure 2).

Thus, the coordinates *x_p_* and *y_p_* in the considered non-conventional coordinate system could be expressed as follows [26]:

(4) $$x_{p}=\frac{\left\|\bar{r}\right\|\cdot \tan(\theta_{x})}{\sqrt{1+\tan^{2}(\theta_{x})+\tan^{2}(\theta_{y})}},$$

(5) $$y_{p}=\frac{\left\|\bar{r}\right\|\cdot \tan(\theta_{y})}{\sqrt{1+\tan^{2}(\theta_{x})+\tan^{2}(\theta_{y})}},$$

Inserting Equations (4) and (5) into Equation (3) gives:

(6) $$\cos(\alpha )=\frac{\tan(\theta_{x})\cdot x+\tan(\theta_{y})\cdot y}{\sqrt{1+\tan^{2}(\theta_{x})+\tan^{2}(\theta_{y})}\cdot \left\|\bar{s}\right\|},$$

In turn, inserting Equation (6) into Equation (2) gives the following dependence between $\left\|\bar{R}\right\|$ and $\left\|\bar{r}\right\|$:

(7) $$\left\|\bar{R}\right\|=\left\|\bar{r}\right\|-\frac{\tan\left(\theta_{x}\right)\cdot x+\tan\left(\theta_{y}\right)\cdot y}{\sqrt{1+\tan^{2}(\theta_{x})+\tan^{2}(\theta_{y})}},$$

A pressure field from the point source at a distance $\left\|\bar{R}\right\|$ can be expressed as follows:

(8) $$p(\left\|\bar{R}\right\|,t)=\frac{P_{0}}{\left\|\bar{R}\right\|}\cdot \exp\left[j\left(wt-k\left\|\bar{R}\right\|\right)\right],$$

The total pressure field at a given point generated by a matrix phased array element can be obtained by integrating the pressure fields generated by point sources along the area of the element. Assuming that the phased array element is a square with side size *a*, the following integral can be written:

(9) $$P(\left\|\bar{R}\right\|,t)=\int_{x-a/2}^{x+a/2}\int_{y-a/2}^{y+y/2}p(\left\|\bar{R}\right\|,t)\;dx\;dy,$$

Carrying out the integration, considering that *a* << *R*, *a* << *r*, and taking into account relation 7 yields the following:

(10) $$\begin{array}{c} P(\left\|\bar{r}\right\|,\theta_{x},\theta_{y},x,y,t)=-\frac{f_{3}^{2}\cdot \exp\left(-ikr\right)\cdot \exp(i\omega t)}{f_{1}\cdot f_{2}\cdot k^{2}\cdot \left\|\bar{r}\right\|}\\ \cdot \left(\exp\left(\frac{ikf_{1}\left(x-a/2\right)}{f_{3}}\right)-\exp\left(\frac{ikf_{1}\left(x+a/2\right)}{f_{3}}\right)\right)\\ \cdot \left(\exp\left(\frac{ikf_{2}\left(y-a/2\right)}{f_{3}}\right)-\exp\left(\frac{ikf_{2}\left(y+a/2\right)}{f_{3}}\right)\right), \end{array}$$

where:

(11) $$\begin{array}{c} f_{1}=\tan(\theta_{x})\\ f_{2}=\tan(\theta_{y})\\ f_{3}=\sqrt{1+\tan^{2}(\theta_{x})+\tan^{2}(\theta_{y})}. \end{array}$$

The synthesized sound pressure can be determined using the following equation:

(12) $$P_{array}(\left\|\bar{r}\right\|,\theta_{x},\theta_{y},t)=\sum_{i=1}^{N}g_{i}\cdot P_{i}(\left\|\bar{r}\right\|,\theta_{x},\theta_{y},x_{i},y_{i},t).$$

where *N* is the number of elements in the matrix phased array and *g* is a binary coefficient. The coefficient is equal to 1 if the element is active in the considered configuration of the sparse array, and it is equal to 0 if the element is excluded from the processes of transmission of ultrasonic waves and reception of echo signals.

**Figure 3:**
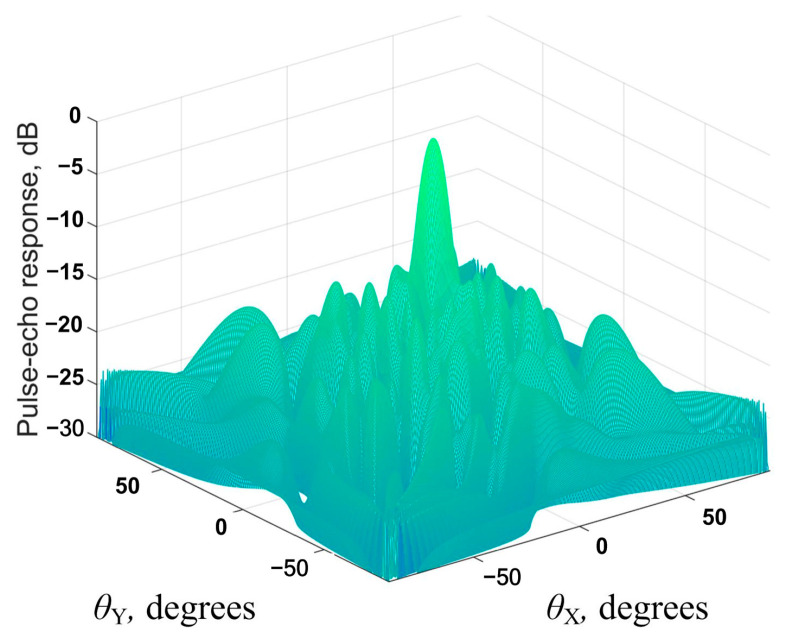
Example of beam directivity diagram.

In this case, the directivity function can be written as follows:

(13) $$H\left(\theta_{x},\theta_{y}\;\right)=\left|\frac{P(\left\|\bar{r}\right\|,\theta_{x},\theta_{y},t)}{p(\left\|\bar{r}\right\|,\theta_{xm},\theta_{ym},t)}\right|,$$

where $\theta_{xm}$ and $\theta_{ym}$ are steering angles. For the TFM, these angles can be assumed as 0. Equation (14) can be used to obtain the beam directivity diagram of the considered matrix phased array configuration. This diagram can be used to evaluate the imaging performance of the considered 2D probe layout. An example of the beam directivity diagram obtained using Equation (13) is shown in Figure 3.

### 2.2. Simulated Annealing Algorithm

**Figure 4:**
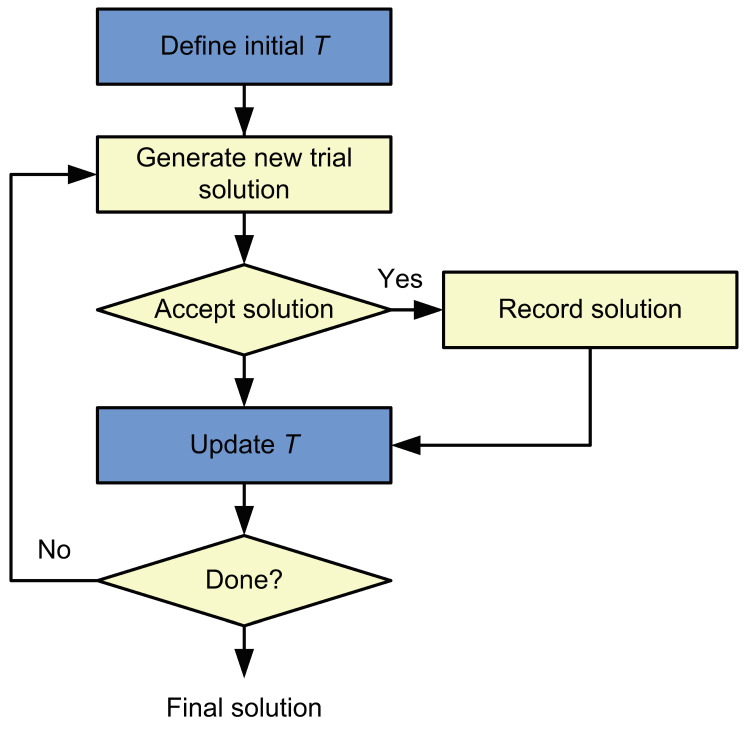
Block diagram of the simulated annealing algorithm.

Simulated annealing is one of the best-known stochastic optimization methods for tasks where the objective function is not explicitly defined. This algorithm can be used to determine the global optima and exclude the selection of local optima as the solution for the optimization task [27]. The basic block diagram of the simulated annealing algorithm is shown in Figure 4. The efficiency of solving optimization problems using simulated annealing depends heavily on the appropriate choice of the parameters of this algorithm. Temperature is one of the most important parameters in simulated annealing used to find the optimal solution to the optimization problems. At the first iterations of the algorithm, the temperature parameter (T) is typically set high. This avoids the selection of local optima as the final solution. As the algorithm runs, this parameter is reduced according to the cooling schedule. The execution of the algorithm is stopped when the temperature parameter reaches the defined limit. The decision to accept the trial state as the current solution is made using the values of the acceptance function.

## 3. Determination of the Optimal Sparse Matrix Phased Array Configuration Using the Simulated Annealing Algorithm

### 3.1. Simulated Annealing Algorithm Implementation

**Figure 5:**
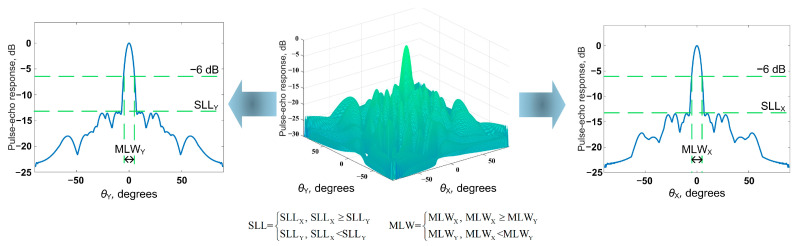
Determination of the beam directivity diagram parameters.

The simulated annealing algorithm was implemented to determine the optimal sparse matrix phased array configurations. At the first stage, a sparse matrix phased array configuration was randomly determined and considered as the current solution. An iterative procedure was then implemented. At each iteration, a new configuration of the sparse matrix phased array was determined, which was considered as a trial solution at this iteration. Then, the beam directivity diagram of the current trial solution was determined using Equation (14), and its parameters were evaluated. The parameters evaluated included the main lobe width (MLW) and the side lobe level (SLL, Figure 5). In this case, the main lobe width was evaluated using the full width at half maximum parameter. The parameters obtained from the beam directivity diagram of the trial configuration of the sparse matrix phased array were then used to evaluate the acceptance function for this configuration and to make a decision regarding its acceptance as the current solution.

The algorithm used for the determination of the sparse matrix phased array configuration was implemented using the MATLAB R2020b software. The algorithm requires test conditions as initial parameters. The test conditions to be defined are as follows:

1. The number of elements in the matrix phased array;
2. The center frequency of the probe elements;
3. The pitch of the array and the dimensions of each element;
4. The velocity of the ultrasonic waves in the test object;
5. The number of active elements in the sparse matrix phased array configuration.

The appropriate choice of simulated annealing parameters is another important factor to determine the efficiency of the optimization solution. During algorithm execution, the temperature parameter was changed from 0.5 to 0.00025. For each constant value of the temperature parameter, five iterations were performed. The temperature parameter changed according to the following law:

(14) $$T_{k+1}=0.98\cdot T_{k}$$

The execution of the algorithm was completed when the temperature parameter reached the lower limit.

The acceptance function is used to make a decision regarding the acceptance of the sparse matrix phased array configuration as the current solution at each iteration of the algorithm execution. This function should be defined with respect to the objective of the optimization task; in this case, the task is to obtain the sparse matrix phased array configuration with a beam directivity diagram that has low SLL and MLW. The acceptance function implemented in the developed algorithm takes the following form:

(15) $$P=\left\{\begin{array}{l} 1;\;\Delta MLW<0,\;\Delta SLL<0\\ \exp(-\Delta MLW/T_{k});\;\Delta MLW>0,\;\Delta SLL<0\;\\ \exp(-\Delta SLL/T_{k});\;\Delta MLW<0,\;\Delta SLL>0\;\\ \exp(-\Delta MLW/T_{k})\cdot \exp(-\Delta SLL/T_{k});\;\Delta MLW>0,\;\Delta SLL>0\; \end{array}\right.$$

where ΔMLW and ΔSLL are the differences in MLW and SLL, respectively, between the beam directivity diagram of the trial configuration of the sparse matrix phased array considered at a current iteration of the algorithm execution and the beam directivity diagram of the current solution.

### 3.2. Results of Simulated Annealing Algorithm Execution

The initial data used to determine the optimal sparse matrix phased array configuration by the developed algorithm are presented in Table 1. These data include the parameters of the probe and the speed of the longitudinal ultrasonic waves in the medium (aluminum).

**Figure 6:**
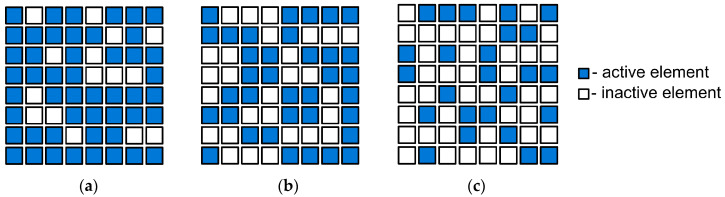
Sparse matrix phased configurations obtained using the developed algorithm: (**a**) sparse matrix phased array configuration with 49 elements; (**b**) sparse matrix phased array configuration with 36 elements; (**c**) sparse matrix phased array configuration with 25 elements.

**Figure 7:**
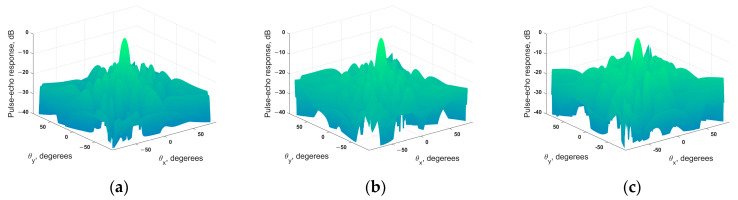
Beam directivity diagrams of sparse matrix phased array configurations obtained using the developed algorithm: (**a**) sparse matrix phased array configuration with 49 elements; (**b**) sparse matrix phased array configuration with 36 elements; (**c**) sparse matrix phased array configuration with 25 elements.

In total, three sparse phased array configurations with 49, 36, and 25 elements were determined using the developed algorithm that employed simulated annealing. These configurations are shown in Figure 6. The beam directivity diagrams of the obtained configurations are shown in Figure 7.

Table 2 shows the parameters of the beam directivity diagrams (MLW and SLL) of the obtained sparse matrix phased array configuration and the full array.

Thus, the directivity diagram parameters of the sparse phased array configurations were close to these of the full matrix phased array. The difference in MLW between all the sparse phased array configurations and the full array did not exceed 0.27 degrees. At the same time, the difference in SLL between the sparse probes and the full array did not exceed 1.51 dB. The obtained differences in the beam directivity diagram parameters of the sparse and full arrays should lead to the restoration of flaw images with a comparable quality.

## 4. Experiments

**Figure 8:**
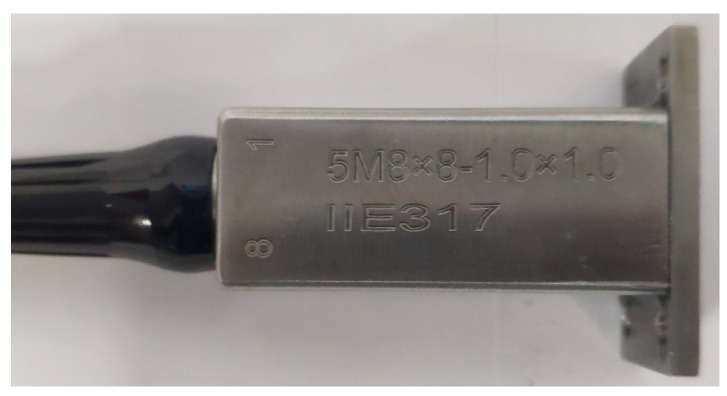
Matrix phased array Doppler 5M8 × 8BP1.0.

The imaging performance of the obtained sparse matrix phased array configurations was verified via in situ experiments in conditions corresponding to the input parameters used during implementation of the simulated annealing algorithm. The experiments employed the matrix phased array Doppler 5M8 × 8BP1.0 (Figure 8). The probe consisted of 64 elements, forming a matrix of 8 × 8 elements. The dimensions of each element were 0.8 × 0.8 mm, and its center frequency was 5 MHz.

**Figure 9:**
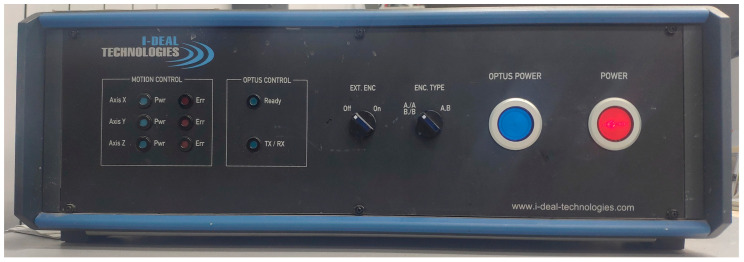
Optus electronic unit.

The Optus electronic unit (I-Deal technologies GmbH, Saarbrücken, Germany, Figure 9) was used for signal acquisition using various configurations of matrix phased arrays. This electronic unit has 128 multiplexed channels, allowing the Doppler 5M8 × 8BP1.0 to be used in FMC and sparse phased array modes of signal acquisition.

**Figure 10:**
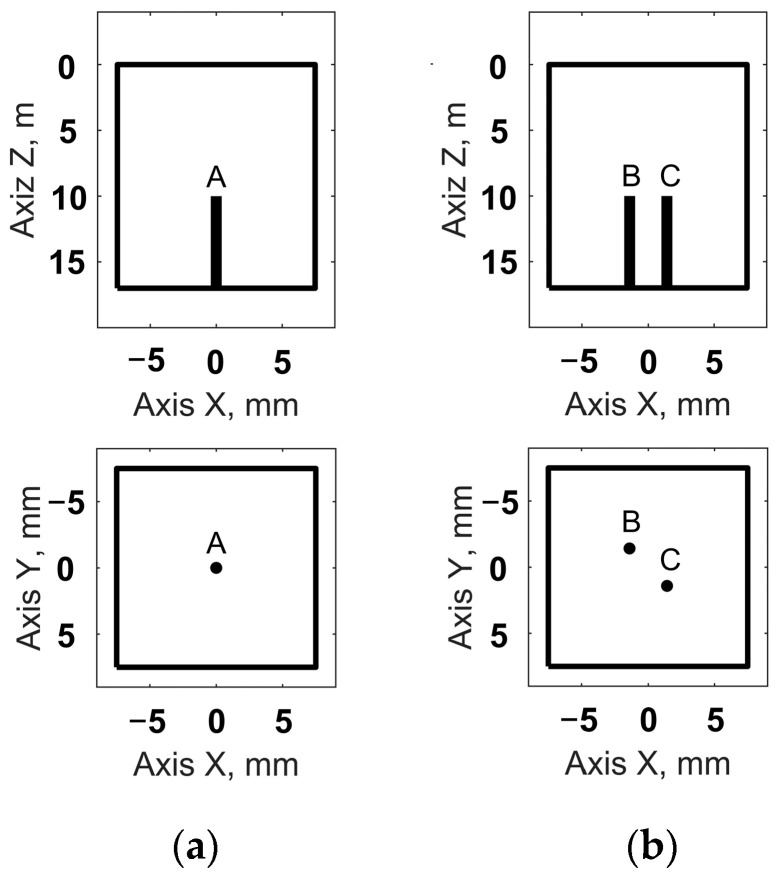
Location of the flaws in the test sections: (**a**) test section 1; (**b**) test section 2.

Two sections of a 17 mm thick aluminum test block were used for experimental verification. Both sections contained flat-bottom holes that were 2 mm in diameter and 10 mm deep. The first section contained one flaw, and the second section contained two flaws located at a distance of 4 mm from each other. The location of the flaws in the test sections is shown in Figure 10.

The experiments considered full array and sparse matrix phased array configurations that were obtained using the developed algorithm. The arrangements of the active elements in the sparse matrix phased array configurations are shown in Figure 6. For all the configurations, the signal acquisition procedure was the same and was based on FMC, taking into account that not all the elements of the probe may be active in the considered transducer configuration. In all the experiments, the probe was initially placed in the center of the test section. The data acquisition was performed in several stages according to the number of active elements in the configuration. At each stage, one active element of the configuration transmitted ultrasonic waves into the specimen, while all the active elements received the reflected signals. This resulted in a set of ultrasonic data that was post-processed. For this purpose, the post-processing algorithm discussed in reference [2] was adapted for volumetric imaging and sparse matrix phased array application.

For all the configurations, the post-processing results were volumetric images of the flaws in the test sections. To evaluate the performances of the different sparse matrix phased array configurations, metrics characterizing the resolution and signal-to-noise ratio were determined. The resolution of the images was evaluated using the array performance indicator (API). This value was calculated for each of the flaws using the following equation [6]:

(16) $$API=\frac{N_{-6\mathrm{dB}}D_{x}D_{y}D_{z}}{\lambda^{3}}$$

where *D_x_*, *D_y_*, and *D_z_* are the dimensions of the voxels along the *X*, *Y*, and *Z* axes, respectively; *N*_−6dB_ is the number of voxels with an amplitude exceeding the −6 dB threshold, where 0 dB corresponds to the maximum obtained amplitude of the flaw; and *λ* is the wavelength of the longitudinal ultrasonic waves in the specimen.

The signal-to-noise ratio can also be evaluated for each of the flaws using the following equation [28]:

(17) $$SNR=20\log_{10}\left(\frac{A_{s}}{A_{n}}\right)$$

where *A_s_* is the maximum amplitude of the signal from the flaw and *A_n_* is the maximum amplitude of the noise at the depth of the flaw location. In the experiments, the value of *A_n_* was obtained by scanning the flawless zone.

The above metrics were used to evaluate the quality of the images obtained using different configurations of matrix phased arrays. According to reference [29], a high quality NDT ultrasonic image should meet the following requirements:

- SNR is higher than 20 dB;
- the level of artifacts relative to the amplitude of the flaw in the image is less than 20 dB;
- the image of the entire flaw boundary is restored.

## 5. Results and Discussion

**Figure 11:**
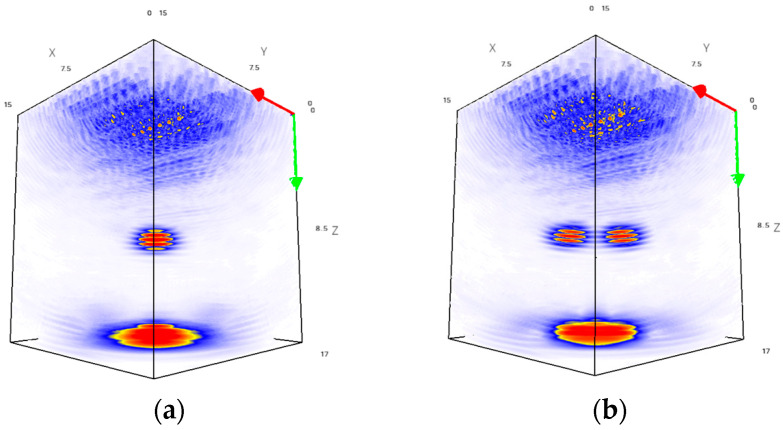
Examples of volumetric results: (**a**) test section 1; (**b**) test section 2.

As mentioned above, the results of ultrasonic imaging performed using different configurations of matrix phased arrays are the volumetric images of the test sections of the aluminum specimens used in the experiments. Examples of such images for both test sections are shown in Figure 11.

**Figure 12:**
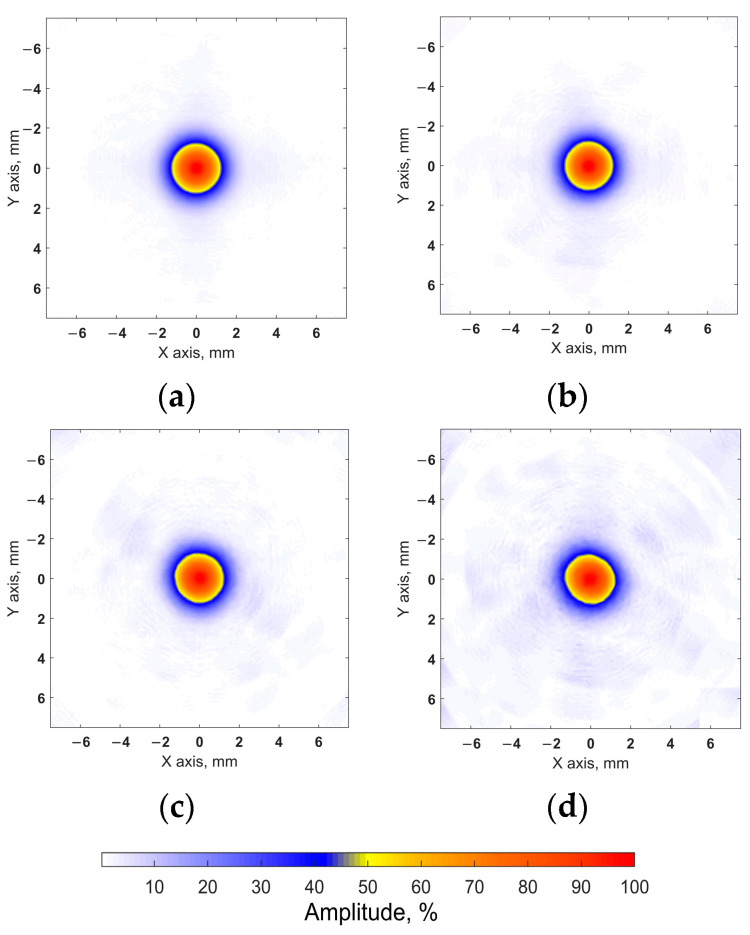
Projections of volumetric images of test section 1 in the XY plane: (**a**) full array; (**b**) 49-element sparse array; (**c**) 36-element sparse array; (**d**) 25-element sparse array.

**Figure 13:**
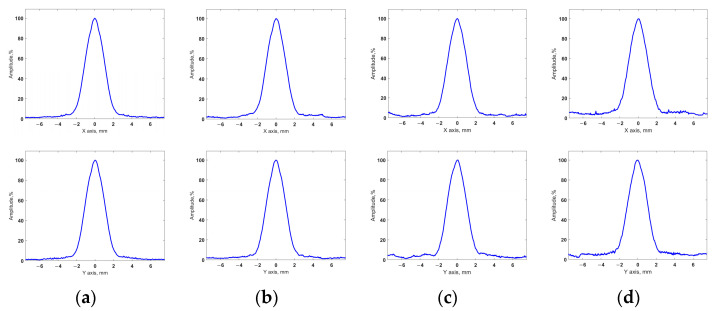
Profiles of obtained volumetric images of test section 1: (**a**) full array; (**b**) 49-element sparse array; (**c**) 36-element sparse array; (**d**) 25-element sparse array.

**Figure 14:**
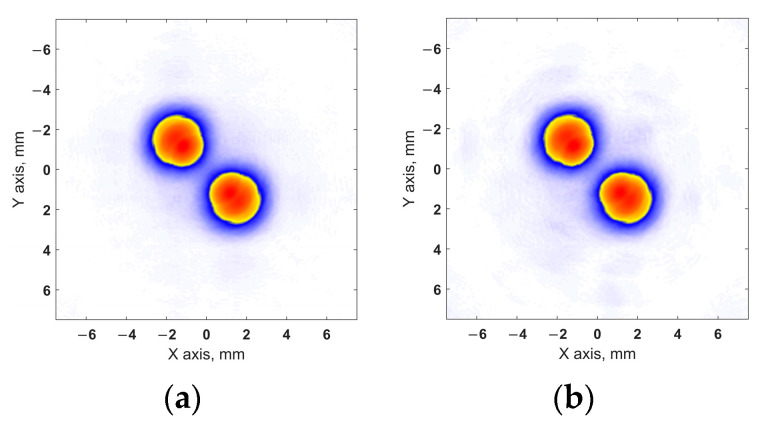

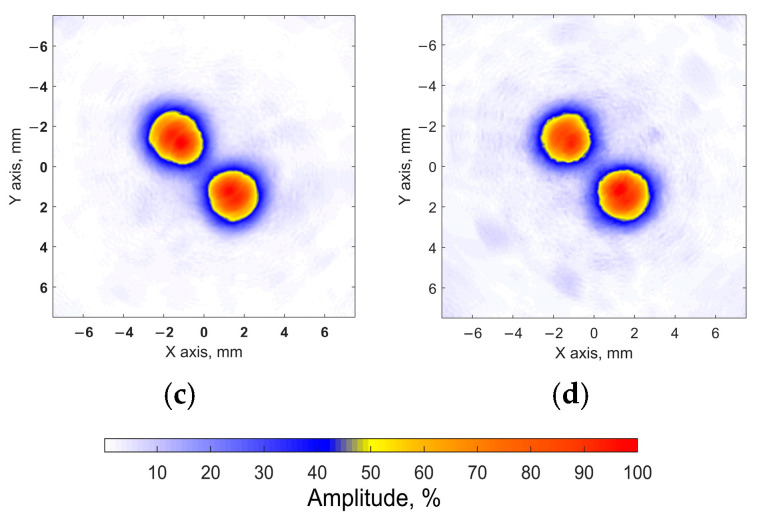
Projections of volumetric images of test section 2 in the XY plane: (**a**) full array; (**b**) 49-element sparse array; (**c**) 36-element sparse array; (**d**) 25-element sparse array.

**Figure 15:**
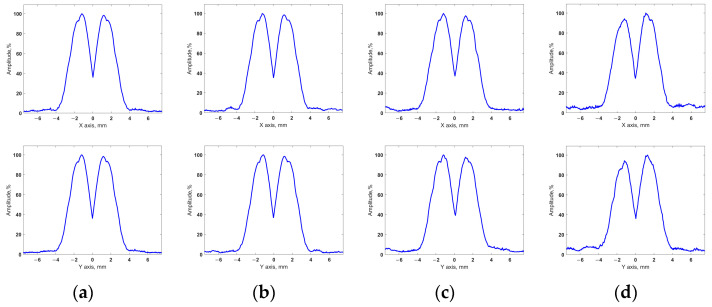
Profiles of obtained volumetric images of test section 2: (**a**) full array; (**b**) 49-element sparse array; (**c**) 36-element sparse array; (**d**) 25-element sparse array.

Figure 12 and Figure 13 show the imaging results for test section 1. Figure 12 shows the projections of the volumetric images obtained using the configurations of sparse arrays on the XY plane. The profiles of these projections on the X and Y axes are shown in Figure 13. Similar results for test section 2 with two closely spaced flat-bottom holes are shown in Figure 14 and Figure 15.

For the obtained results, the API and SNR for each of the flaws were evaluated using Equations (16) and (17). The resulting values are presented in Table 3, where the flaws are indicated, similar to the flaws in Figure 10. Furthermore, the obtained API and SNR for the flaws in the images that were restored using the sparse array data were compared with the corresponding values for the flaws in the images obtained using the full matrix phased array.

Thus, the images that were restored using the sparse matrix phased array configurations had a resolution that was close to that of the images obtained using the full matrix array. The difference in the API between the considered cases did not exceed 3.44%. Furthermore, all the sparse matrix phased array configurations used were able to resolve closely spaced flaws in test section 2 using the −6dB drop method. The SNR of all the results obtained with sparse probes was greater than 20 dB, and these images did not contain artifacts with amplitudes exceeding −20 dB relative to the maximum amplitude of the flaw. The obtained results included high-quality images of both test sections for all the applied sparse arrays configurations. At the same time, the application of sparse arrays reduced the amount of ultrasonic data compared to the full array. In our experiments, the use of 49-, 36-, and 25-element sparse arrays reduced the set of signals 1.7-, 3.1-, and 6.6-fold, respectively. The obtained results demonstrate a high imaging performance of the resulting sparse matrix phased array configurations and the efficiency of the proposed approach for determining these layouts with regard to the conditions of ultrasonic testing.

## 6. Conclusions

TFM/FMC provides high imaging performance, but it is time consuming. This paper proposed a method to design a sparse matrix phased array using the simulated annealing algorithm for TFM imaging. Within the framework of the considered approach, the parameters of the beam directivity diagram were optimized in order to determine the appropriate matrix phased array layout. The beam directivity diagrams were evaluated in the modified spherical coordinate system using far-field approximation.

The considered approach was applied for proper conditions of TFM imaging. As a result, sparse matrix phased array configurations with 49, 36, and 25 elements were determined. The parameters of the beam directivity diagrams of these sparse phased arrays were close to the corresponding parameters of the full array. The difference in the main lobe width for the above configuration did not exceed 0.27 degrees compared to the same parameters for the 64-element full matrix phased array when all the probe elements were active. A similar comparison performed for the side lobe level showed that the maximum difference between the obtained sparse arrays and the full array was 1.51 dB.

The performance of the obtained sparse matrix phased array configurations was evaluated via in situ experiments. The experimental conditions corresponded to the ultrasonic imaging parameters considered for the determination of the sparse matrix phased array configuration using the developed method. As a result, high-resolution images were obtained for both test sections with flat-bottom holes using the obtained sparse matrix phased array configurations. The difference in the API between the results obtained using sparse matrix phased arrays and the full array did not exceed 3.44% for all the cases considered. In addition, in all the images obtained for test section 2, the closely spaced defects were resolved using the −6dB drop method. This is another indicator of the achieved resolution of the restored images. In addition, the SNR for the flaws in the obtained images was greater than 20 dB, with no artefacts of amplitudes exceeding −20 dB found in the results. The evaluated quality metrics of the volumetric images demonstrate the high quality of the results obtained using all the determined sparse matrix phased array configurations and the efficiency of the proposed sparse matrix phased array design method. At the same time, the use of sparse matrix phased arrays allows for a significant reduction in the volume of data to be processed compared to the full array. The number of signals was reduced 1.7-fold (49 active elements), 3.1-fold (36 active elements), and 6.6-fold (25 elements) for the sparse matrix phased arrays. In practice, this should significantly reduce the ultrasonic imaging time.

The results obtained demonstrate the efficiency of the proposed approach and serve as a basis for further research and development. The next stage of research should be focused on the effects of the sparse phased array on the imaging time during post-processing with parallelization of calculations using GPU or FPGA. Furthermore, the adaptation of the developed design method to ultrasonic imaging of complex-shaped objects can also be considered.

## Figures and Tables

**Table 1:** Matrix phased array parameters for which the optimization task was solved.

Parameter	Value
Number of elements	8 × 8
Center frequency, MHz	5
Pitch, mm	1
Element dimensions, mm	0.8 × 0.8 mm
Speed of ultrasonic waves in the media	6350 m/s

**Table 2:** Parameters of beam directivity diagrams of all the considered configurations of the matrix phased arrays.

Number of Elements in Configuration	MLW, Degrees	SLL, dB
64	10.88	−13.23
49	10.96	−12.53
36	11.13	−13.29
25	11.15	−11.72

**Table 3:** API and SNR values for the obtained results.

Configuration	Defect	API		SNR	
		Value	Difference, %	Value, dB	Difference, dB
Full array	A	0.623	0	36.21	0
	B	0.649	0	33.89	0
	C	0.668	0	33.18	0
Sparse 49-element	A	0.614	1.44	33.49	−2.72
	B	0.640	1.39	32.71	−1.18
	C	0.670	0.30	30.68	−2.5
Sparse 36-element	A	0.620	0.48	32.61	−3.6
	B	0.661	1.85	29.67	−4.22
	C	0.650	2.69	29.48	−3.7
Sparse 25-element	A	0.629	0.96	27.98	−8.23
	B	0.634	2.31	26.35	−7.54
	C	0.645	3.44	26.83	−6.35

## Data Availability

The data that support the findings of this study are available from the corresponding author upon reasonable request.
